# A pleuromutilin–ciprofloxacin hybrid exhibits potent activity against *Streptococcus suis*

**DOI:** 10.3389/fcimb.2026.1920241

**Published:** 2026-08-14

**Authors:** Guiyu Zhou, Feike Zhao, Xirui Jia, Xiaoyu Wang, Cunchun Dai, Liangzhu Chen, Binghu Fang

**Affiliations:** 1 College of Veterinary Medicine, South China Agricultural University, Guangzhou, Guangdong, China; 2 Guangdong Wenshi Dahuanong Biotechnology Co, Ltd, Yunfu, Guangdong, China

**Keywords:** antibacterial mechanism, antimicrobial candidate, antimicrobial resistance, hybrid antibacterial agent, Streptococcus suis

## Abstract

The emergence and dissemination of antimicrobial resistance in *Streptococcus suis*, an important zoonotic pathogen, have created an urgent demand for new therapeutic agents with improved antibacterial efficacy. To address this challenge, we previously designed and synthesized a novel hybrid molecule, PHC, by linking ciprofloxacin to a pleuromutilin derivative via an L-homocysteine bridge. Although preliminary evaluations indicated favourable antibacterial activity, the antimicrobial effect of PHC against *S. suis* and its mechanism of action have not yet been systematically characterized. Therefore, the present study aimed to investigate the antibacterial activity of PHC against *S. suis* and elucidate its underlying antibacterial mechanisms.

**Methods:**

The antibacterial efficacy of PHC was assessed through time-kill kinetics and a murine infection model. Molecular docking was conducted to predict the interactions between PHC and its potential molecular targets, and RT–qPCR analysis was performed to evaluate the effects of PHC on genes associated with DNA replication, ribosomal function, oxidative stress, and energy metabolism in *S. suis*. Biofilm formation was quantified using crystal violet staining. In addition, DAPI/PI staining and DiSC3(5) fluorescence assays were employed to assess bacterial membrane integrity and membrane potential, respectively. Changes in intracellular ATP levels and ROS production were further measured to characterize the antibacterial mode of action of PHC.

**Results:**

PHC exhibited potent antibacterial activity against *S. suis* both *in vitro* and *in vivo*. Molecular docking and transcriptional analyses suggested that PHC may interfere with bacterial DNA replication and protein synthesis by affecting topoisomerase- and ribosome-associated pathways. In addition, PHC disrupted membrane integrity and membrane potential, altered cellular energy metabolism and promoted oxidative stress responses. Furthermore, PHC demonstrated favourable therapeutic efficacy and biosafety in a murine infection model.

**Conclusion:**

PHC exhibits potent antibacterial activity against *S. suis* through interference with DNA replication- and protein synthesis-related processes, accompanied by enhanced bacterial stress responses and disruption of membrane integrity and cellular homeostasis. This study provides a theoretical basis for the further development of PHC and offers valuable insights for the design of novel hybrid antibacterial agents against *S. suis*.

## 1 Introduction

*Streptococcus suis* (*S. suis*) is an encapsulated gram-positive pathogen that typically appears as spherical or ovoid cells arranged in pairs or short chains (Haas and Grenier, 2018). On the basis of the antigenic diversity of its capsular polysaccharides (CPS), *S. suis* can be classified into multiple serotypes (Gottschalk et al., 2010; Hildebrand et al., 2010). Among these serotypes, serotype 2 (SS2) is considered the most virulent and clinically significant, accounting for the majority of *S. suis* infections in both swine and humans worldwide (Hildebrand et al., 2010; Dong et al., 2025).

*Streptococcus suis* is a major zoonotic pathogen of global significance, causing substantial economic losses in the swine industry and posing a serious threat to human health (Huong et al., 2014). In pigs, *S. suis* commonly colonizes the upper respiratory tract and can lead to a range of invasive diseases, including pneumonia, endocarditis, arthritis, meningitis, and septicemia (Huong et al., 2014; Lunha et al., 2022). In humans, infection is most frequently associated with meningitis and sepsis and can, in severe cases, progress to streptococcal toxic shock-like syndrome (STSLS), which is characterized by a high mortality rate (Huong et al., 2014; Bai et al., 2024). This pathogen is widely distributed worldwide, particularly in Southeast Asia, and is recognized as one of the most important bacterial pathogens in modern swine production.

Antimicrobial therapy remains the primary strategy for controlling *Streptococcus suis* infections, with β-lactams, macrolides, and tetracyclines being among the most commonly used agents. Nevertheless, the widespread emergence of resistance to macrolides and tetracyclines has substantially compromised their clinical efficacy. Resistance mechanisms in *S. suis* include alterations in penicillin-binding proteins, ribosomal protection, active efflux, and target-site modification, which collectively contribute to reduced susceptibility to multiple antimicrobial classes. Consequently, *S. suis* has been recognized as an important reservoir of antimicrobial resistance genes, highlighting the urgent need for the development of novel antibacterial agents with improved efficacy against resistant strains (Cuong et al., 2018; Liu et al., 2025).

Since the isolation of pleuromutilin in 1951, extensive attention has been devoted to its structural optimization and the development of semi-synthetic derivatives. However, the structural complexity of pleuromutilin derivatives presents challenges for chemical modification and synthesis, and several compounds exhibit poor aqueous solubility, which may limit their bioavailability and clinical development potential (Schlünzen et al., 2004; Goethe et al., 2019). In veterinary medicine, the development of pleuromutilin derivatives has been relatively limited, with tiamulin and valnemulin being the major commercially available representatives. Owing to their unique antibacterial mechanism involving binding to the peptidyl transferase center (PTC) of the 50S ribosomal subunit and inhibition of bacterial protein synthesis, pleuromutilin antibiotics exhibit a relatively low propensity for cross-resistance and retain potent activity against certain resistant Gram-positive pathogens, highlighting their considerable potential for further development (Goethe et al., 2019; Liu et al., 2026).

Quinolones are a class of broad-spectrum antibacterial agents that exert their effects primarily by targeting bacterial DNA gyrase and topoisomerase IV, thereby inhibiting DNA replication and cell division (Hooper and Jacoby, 2016). However, the extensive clinical use of quinolones has led to the rapid emergence and dissemination of resistance. The major mechanisms of quinolone resistance include chromosomal mutations in target genes such as *gyrA* and *parC*, plasmid-mediated quinolone resistance, and the overproduction of multidrug efflux pumps (Escudero et al., 2007).

The development of novel antibacterial agents remains a critical strategy for combating antimicrobial resistance. In recent years, hybrid drug design has attracted considerable attention because it offers the potential to enhance antibacterial efficacy while simultaneously modulating multiple bacterial targets. Owing to the structural versatility of the quinolone scaffold and the availability of several modification sites, quinolones have become attractive building blocks for the design of hybrid antibacterial agents (Lungu et al., 2022). Consequently, quinolone-based hybrids have emerged as a promising direction in antibacterial drug discovery. Nevertheless, the increased structural complexity of hybrid molecules often results in lengthy synthetic routes, reduced overall yields, and increased production costs. In addition, their relatively large molecular size may adversely affect physicochemical properties, including aqueous solubility and linker stability. As a result, the majority of quinolone-based hybrid molecules remain at the preclinical research stage (Pokrovskaya and Baasov, 2010).

Drug conjugation represents an attractive strategy for antibacterial drug development by covalently integrating distinct pharmacophores into a single molecular framework, thereby potentially increasing biological activity and overcoming existing resistance mechanisms (Gupta and Datta, 2019).

**Figure 1:**
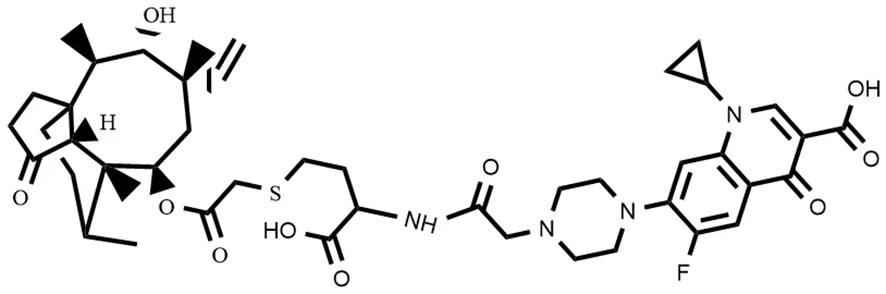
Structure of hybrid compound PHC.

N-Acetylcysteine possesses several advantageous structural features, including a thiol group, the ability to serve as a precursor of glutathione (GSH), and the potential to function as a cysteine-derived linker in cleavable conjugation systems (Navath et al., 2008), and has also been utilized as a bridging linker in drug conjugation strategies (Gok et al., 2025). Given the high structural similarity such as common amino acid scaffold and a reactive thiol moiety between N-acetylcysteine and L-homocysteine (Nyui et al., 2019), we speculated that L-homocysteine may possess similar structural advantages. Therefore, we innovatively selected L-homocysteine as a linker bridge for the conjugation of a truncated pleuromutilin derivative with ciprofloxacin to construct the hybeid molecule PHC (**Figure 1**).

Preliminary studies revealed that PHC possessed potent antibacterial activity against gram-positive bacteria, particularly *Streptococcus suis*. Nevertheless, its antibacterial properties and mechanism of action remain poorly understood.

Therefore, the present study aimed to evaluate the antibacterial efficacy of PHC against *S. suis in vitro* and *in vivo*, investigate its effects on bacterial membrane function and cellular physiology, and assess its biosafety. This study provides mechanistic insights into the antibacterial activity of PHC and supports the further development of hybrid antibacterial agents to combat *S. suis* infections.

## 2 Methods

### 2.1 Bacterial strains and culture conditions

*Streptococcus suis* strains were routinely cultured in brain heart infusion (BHI) medium. For long-term storage, fresh bacterial colonies were suspended in sterile BHI broth supplemented with 30% (v/v) glycerol and preserved at −80 °C. Prior to each experiment, frozen stocks were streaked onto BHI agar plates supplemented with 10% (v/v) porcine serum and incubated at 37 °C for 24 h. The recovered bacteria were passaged twice to ensure viability and phenotypic stability. A single colony was subsequently inoculated into BHI broth and cultured statically at 37 °C for 11.5 h for experimental use.

### 2.2 Determination of minimum inhibitory concentration

The minimum inhibitory concentration (MIC) of PHC against *Streptococcus suis* was determined by the broth microdilution method in accordance with Clinical and Laboratory Standards Institute (CLSI) guidelines. Bacterial suspensions were prepared in BHI broth and adjusted to approximately 1 × 10^6^ CFU/mL. Wells containing bacteria without antimicrobial treatment served as growth controls, while wells containing sterile medium alone served as sterility controls. After incubation at 37 °C for 20–24 h, the MIC was recorded as the lowest concentration of PHC that completely prevented visible bacterial growth. Each experiment was independently repeated three times, and duplicate plates were analysed for each concentration.

### 2.3 Time–kill curve assay

An overnight culture of *Streptococcus suis* SS-27 was diluted with fresh sterile BHI broth to approximately 1 × 10^6^ CFU/mL. PHC, ciprofloxacin, and tiamulin were added separately to obtain final concentrations of 0.5×, 1×, 2×, 4×, and 8× MIC. The untreated control group received an equivalent volume of PBS, and the final reaction volume was maintained at 3 mL.

The cultures were incubated at 37 °C, and samples were collected at 0, 2, 4, 6, 8, 10, and 12 h. Following serial dilution, aliquots were plated onto BHI agar plates and incubated for bacterial enumeration. Viable counts were determined as CFU/mL and expressed as log10 CFU/mL for the construction of the time–kill curves. Each concentration is repeated three times, and the experiment is repeated three times.

### 2.4 Determination of mutant prevention concentrations

The mutant prevention concentrations(MPCs) of PHC, ciprofloxacin, and tiamulin were determined as previously described with minor modifications. Antimicrobial agents were incorporated into serum-supplemented BHI agar at concentrations ranging from 1× to 32× MIC using twofold serial dilutions. A high-density bacterial suspension (approximately 1 × 10^9^ CFU/mL) was prepared, and 100 μL was spread uniformly onto agar plates containing the indicated drug concentrations. Following incubation at 37 °C for 72 h, the mutant prevention concentration(MPC) was recorded as the lowest concentration at which no visible colonies were detected. Each experiment was independently repeated three times, and duplicate plates were analysed for each concentration.

### 2.5 Safety assessment

#### 2.5.1 Evaluation of cytotoxicity by a CCK-8 assay

The cytotoxic effects of PHC on HEK293T cells were assessed using a Cell Counting Kit-8 (CCK-8) assay. Ciprofloxacin and tiamulin were included as comparator drugs. HEK293T cells in the exponential growth phase were seeded into 96-well plates at a density of 1 × 10^4^ cells/well and cultured overnight. Serial twofold dilutions of each compound were prepared in complete medium, with a maximum concentration of 64 μg/mL. Cells were exposed to the indicated concentrations for 24 h at 37 °C under 5% CO_2_.

Following treatment, CCK-8 reagent was added according to the manufacturer’s instructions, and the plates were further incubated before the absorbance was measured at 450 nm. Wells containing untreated cells and medium alone served as the cell control and blank control groups, respectively. All experiments were conducted in triplicate. The cell viability is expressed as a percentage relative to that of the untreated control.

#### 2.5.2 Haemolysis assay

The haemolytic activity of PHC was assessed using defibrinated sheep red blood cells (RBCs). Sheep blood was centrifuged at 1,000 rpm for 10 min at 4 °C, after which the erythrocytes were washed three times with sterile PBS before being resuspended in an 8% (v/v) RBC suspension.

Serial dilutions of filter-sterilized PHC were prepared to yield final concentrations ranging from 1 to 64 μg/mL. PHC solutions were mixed with the RBC suspension at a ratio of 9:1 (v/v) and incubated at 37 °C for 1 h. PBS and 0.2% Triton X-100 served as the negative and positive controls, respectively. After centrifugation, the absorbance of the supernatants was determined at 576 nm using a microplate reader. The haemolysis rate was calculated according to the following equation:

Haemolysis (%) = [(OD_sample − OD_negative control)/(OD_positive control − OD_negative control)] × 100. All the experiments were performed independently in triplicate.

### 2.6 Biofilm antiformation assay

The inhibitory effect of PHC on *Streptococcus suis* SS-27 biofilm formation was assessed by crystal violet staining. Overnight cultures were diluted 1:100 in fresh BHI broth and exposed to PHC at 0.5×, 1×, 2×, and 4× the MIC. Untreated cultures receiving an equal volume of PBS served as controls.

Following incubation at 37 °C for 24 h in 96-well plates, planktonic cells were removed, and the attached biofilms were fixed with methanol, stained with 0.1% crystal violet, and washed with PBS. The retained stain was subsequently dissolved in 33% glacial acetic acid, and the biofilm biomass was quantified by measuring the absorbance at 590 nm. Representative images were obtained after staining. All the assays were conducted in triplicate.

### 2.7 Membrane integrity assay

Changes in bacterial membrane integrity following PHC treatment were assessed using DAPI/PI dual fluorescence staining. Overnight cultures of *Streptococcus suis* SS-27 were diluted 1:100 in fresh BHI broth and exposed to PHC at 0.5× and 1× the MIC. Untreated bacteria receiving an equivalent volume of PBS served as controls.

After incubation at 37 °C for 6 h, the bacterial cells were collected by centrifugation (4,000 rpm, 3 min, 4 °C), washed three times with PBS, and stained with DAPI and PI for 10 min in the dark. Excess dye was removed by washing with PBS, and the stained cells were resuspended and examined using an inverted fluorescence microscope. Representative fluorescence images were acquired for subsequent analysis.

### 2.8 Membrane potential assay

Changes in the membrane potential of *Streptococcus suis* SS-27 following PHC treatment were determined using the membrane potential-sensitive probe DiSC3(5). The bacterial suspensions were adjusted to 1 × 10^8 CFU/mL and transferred to a 96-well microplate at 160 μL per well. After the addition of DiSC3(5) according to the manufacturer’s protocol(Beyotime Biotechnology, China), the samples were incubated in the dark at 37 °C for 25 min to stabilize the fluorescence signal.

Baseline fluorescence was recorded for 10 min (Ex = 622 nm, Em = 670 nm). PHC was then added to final concentrations of 0.5×, 1×, 2×, and 4× MIC, while the PBS-treated bacteria served as controls, every concentration repeated three times and test repeated three times. The fluorescence intensity was monitored continuously for 30 min. An increase in fluorescence intensity was interpreted as membrane depolarization. The resulting fluorescence kinetics were plotted as the fluorescence intensity versus time.

### 2.9 Protein leakage assay

The leakage of intracellular proteins from *Streptococcus suis* SS-27 following PHC treatment was quantified using a BCA protein assay. Bacterial suspensions (1 × 10^8 CFU/mL) were exposed to PHC at 0.5×, 1×, 2×, and 4× the MIC, while PBS-treated bacteria served as controls. The samples were incubated at 37 °C, and the culture supernatants were collected at designated time points (0, 2, 4, 6, 8, 12, and 24 h) following centrifugation (4,000 rpm, 5 min, 4 °C).

The protein concentrations in the supernatants were determined using BCA protein assay kit according to the manufacturer’s protocol(Beyotime Biotechnology, China). The absorbance was recorded at 562 nm, and protein concentrations were calculated using a standard curve. All experiments were conducted in triplicate. Increased extracellular protein levels were interpreted as evidence of increased membrane permeability and cellular leakage.

### 2.10 ATP quantification assay

Intracellular ATP levels in *Streptococcus suis* SS-27 were measured following PHC treatment using a bioluminescence-based ATP assay kit(Beyotime Biotechnology, China). Bacterial suspensions (1 × 10^8 CFU/mL) were exposed to PHC at 0.5×, 1×, 2×, and 4× MIC for 6 h at 37 °C, while the PBS-treated bacteria served as controls.

After incubation, the bacterial cells were harvested, washed three times with PBS, and lysed with 200 μL of lysis buffer. The cell lysates were centrifuged (13,000 × g, 5 min, 4 °C), and the supernatants were collected for ATP analysis. ATP-dependent luminescence was measured according to the manufacturer’s protocol, and ATP concentrations were determined from a standard calibration curve. The results were used to evaluate the impact of PHC on bacterial energy metabolism. All experiments were conducted in triplicate.

### 2.11 Measurement of intracellular ROS levels

The intracellular ROS levels of *Streptococcus suis* SS-27 following PHC treatment were assessed using a DCFH-DA-based ROS detection kit (Beyotime Biotechnology, China). Bacterial suspensions (1 × 10^8 CFU/mL) were incubated with the fluorescent probe at 37 °C for 20 min in the dark and gently mixed at regular intervals to facilitate probe uptake. Excess probe was removed by washing the cells three times with PBS after centrifugation (4,000 rpm, 3 min, 4 °C).

The stained bacterial suspensions were transferred to 96-well plates and treated with PHC at final concentrations of 0.5×, 1×, 2×, and 4× MIC. PBS-treated bacteria served as controls. After incubation for 1 h at 37 °C, the fluorescence intensity was measured at excitation and emission wavelengths of 488 nm and 525 nm, respectively. Increased fluorescence intensity was interpreted as elevated intracellular ROS accumulation. All experiments were performed independently in triplicate under light-protected conditions.

### 2.12 Quantification of metabolism-related genes by RT–qPCR

To investigate the transcriptional response of *Streptococcus suis* to PHC treatment, RT–qPCR analysis was performed on genes involved in DNA replication (*gyrA* and *parC*), ribosomal function (*23S rRNA*), stress responses (*recA* and *nox*), and energy metabolism (*ldh* and *atpD*). Bacterial cultures were exposed to sub-MIC concentrations (0.5× MIC) of PHC, ciprofloxacin, or tiamulin for 6 h, while PBS-treated cultures served as untreated controls.

Total RNA was isolated from harvested bacterial pellets and reverse-transcribed into cDNA. Quantitative PCR was subsequently performed using gene-specific primers, with *16S rRNA* used as the internal reference gene. Relative transcript levels were calculated using the comparative Ct (2^-ΔΔCt) method. All experiments were conducted in triplicate.

### 2.13 Anti-haemolytic activity assay

The anti-haemolytic activity of PHC was evaluated using a sheep erythrocyte haemolysis assay. Briefly, overnight cultures of *Streptococcus suis* SS-27 (1 × 10^8 CFU/mL) were centrifuged at 10,000 rpm for 2 min at 4 °C, after which the culture supernatants were collected. Defibrinated sheep erythrocytes were washed three times with sterile phosphate-buffered saline (PBS) and resuspended in an 8% (v/v) suspension. The bacterial supernatant was mixed with the erythrocyte suspension at a 1:1 ratio and incubated at 37 °C for 1 h. PHC was subsequently added to achieve final concentrations of 0.5×, 1×, 2×, and 4× MIC, while an equal volume of PBS was added to the positive control group. After further incubation at 37 °C for 1 h, the mixtures were centrifuged at 3,000 × g for 10 min at 4 °C, and the absorbance of the supernatants was measured at 540 nm using a microplate reader. Erythrocytes incubated with PBS alone served as the negative control. Haemolytic activity was assessed on the basis of haemoglobin release, and all experiments were performed in triplicate.

### 2.14 *In Vivo* studies

#### 2.14.1 Survival analysis

Physiologically healthy mice with comparable body weights were randomly divided into the following five groups using completely randomized design (n = 12 per group; equal numbers of males and females): the PHC, ciprofloxacin, tiamulin, infected untreated model, and uninfected control groups. Following acclimation of three days, the infected mice were challenged via tail vein injection with 0.1 mL of *S. suis* SS-27 suspension (5 × 108 CFU/mL). One hour post-infection, the treatment groups received PHC, ciprofloxacin, or tiamulin via oral gavage at 40 mg/kg every 12 h for 72 h and untreated model group, uninfected control group separately received vehicle solution and normal saline. Survival was recorded from the time of the first treatment administration until the end of the observation period.

#### 2.14.2 Evaluation of *In Vivo* antibacterial activity in a murine bacteremia model

The *in vivo* antibacterial efficacy of PHC was evaluated using a murine bacteremia model. Thirty male and thirty female mice with healthy and comparable body weights were randomly assigned via completely randomized design to five groups with six males and six females in each group (n = 12 per group): the PHC treatment group, ciprofloxacin treatment group, tiamulin treatment group, infected untreated model group, and uninfected control group. After three days of acclimation, mice in the infected groups were challenged via tail vein injection with 0.1 mL of a *Streptococcus suis* SS-27 suspension (5 × 10^8^ CFU/mL). One hour post-infection, PHC, ciprofloxacin, or tiamulin was administered by oral gavage at a dosage of 40 mg/kg body weight every 12 h for 48 h. Mice in the model group received an equivalent volume of vehicle solution and uninfected control group received normal saline.

Twelve hours after the final treatment, the mice were anaesthetized for blood collection and subsequently euthanized. The heart, liver, spleen, lungs, and kidneys were aseptically harvested. The tissue samples were homogenized, serially diluted, and plated on BHI agar for bacterial burden determination, while parallel tissue samples were processed for histopathological examination.

### 2.15 Molecular docking analysis

To explore the potential binding interactions of PHC with bacterial targets, molecular docking studies were performed. The ternary complex of *Streptococcus pneumoniae* topoisomerase IV (ParC–ParE) with DNA and clinafloxacin (PDB ID: 3RAD), the *Escherichia coli* DNA gyrase–DNA cleavage complex (PDB ID: 6RKV), and the *Staphylococcus aureus* 50S ribosomal subunit in complex with lefamulin (PDB ID: 5HL7) were selected as receptor structures (Laponogov et al., 2009; Saleh et al., 2022; Yi et al., 2025).

Protein structures were obtained from the Protein Data Bank (PDB), after which water molecules were removed and polar hydrogen atoms were added using AutoDock Tools. Docking simulations were conducted using AutoDock4.2.6, the docking grid box was centered on the native ligand-binding site with dimensions of 30*30*30 Å, and binding conformations were visualized and analysed using PyMOL 2.5.5(Schrödinger, New York, NY, USA).Schematic two-dimensional representations of ligand-protein interactions were generated using LigPlot+ 2.3.1(European Molecular Biology Laboratory-European Bioinformatics Institute,Cambridge, UK).

### 2.16 Statistical analysis

Statistical analyses were conducted using GraphPad Prism software (9.5.0), and one-way ANOVA was used followed by Tukey’s *post hoc* test for multiple comparisons. Statistical significance was set at *p< 0.05, **p< 0.01, ***p< 0.001, and ****p< 0.0001.

### 2.17 Animal ethics statement

All animal experiments were carried out in strict accordance with the guidelines for the care and use of laboratory animals. The study was reviewed and approved by the Animal Ethics Committee (Approval Number: 2026C029).

## 3 Results

### 3.1 Minimum inhibitory concentration and time–kill curve

The MIC of PHC against *Streptococcus suis* SS-27 was 0.125 μg/mL, whereas the MICs of tilmicosin and ciprofloxacin were 8 μg/mL and 16 μg/mL, respectively. On the basis of the CLSI interpretive criteria, SS-27 was classified as resistant to ciprofloxacin.

**Figure 2:**
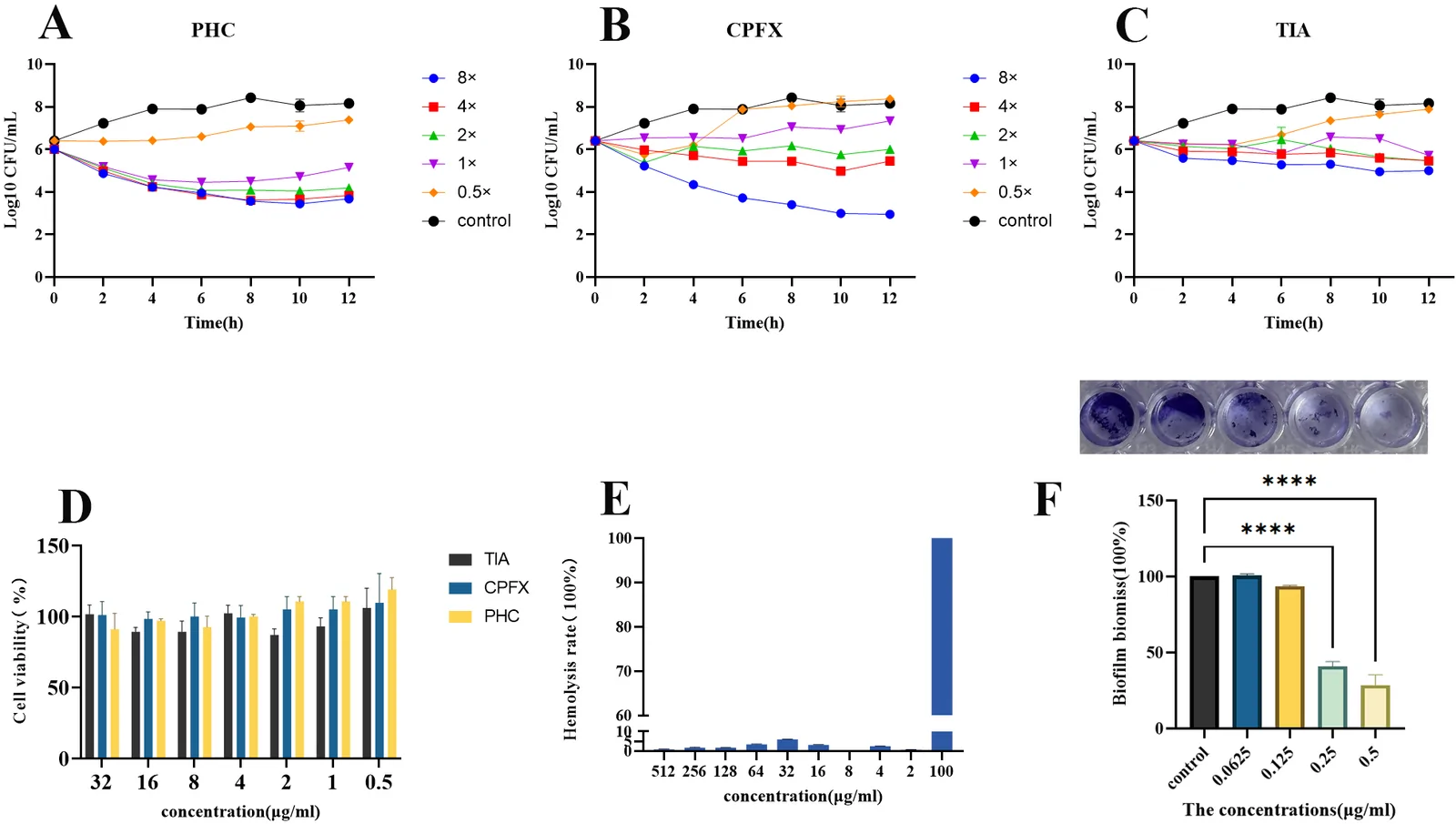
**(A–C)** Time–kill curve of PHC, ciprofloxacin(CPFX), and tiamulin(TIA) against SS-27. **(D)** Cytotoxicity assay: Cell viability at different concentrations of PHC determined using the CCK-8 assay. **(E)** Haemolysis assay: Hemolytic activity of PHC against sheep erythrocytes at different concentrations. **(F)** Anti-biofilm formation test by crystal violet staining, Statistical analysis was performed using one-way ANOVA followed by multiple comparisons, ****p < 0.0001.

Time-kill assays demonstrated the antibacterial activity of PHC against SS-27 (**Figure 2**). PHC significantly reduced bacterial viability at 1×, 2×, and 4× the MIC, the antibacterial activity was not significantly concentration-dependent. Ciprofloxacin exhibited antibacterial activity only at 8× MIC, whereas treatment at 4× MIC resulted in a limited reduction of approximately 1.5 Log10 CFU/mL, with no apparent antibacterial effect observed at the other tested concentrations. Tiamulin showed a weaker bactericidal effect, reducing SS-27 viability by a maximum of only 1.5 Log10 CFU/mL at 8× MIC. Compared with ciprofloxacin and tilmicosin, PHC consistently maintained lower bacterial counts across all the tested concentrations throughout the observation period.

### 3.2 Mutation selection window

The mutation selection window (MSW) is defined as the concentration range between the minimum inhibitory concentration (MIC) and the mutant prevention concentration (MPC) and can be expressed using the selection index (SI = MPC/MIC). A lower SI value reflects a narrower MSW.

The MPCs of PHC, tiamulin, and ciprofloxacin against SS-27 were 1 μg/mL (8× MIC), 64 μg/mL (8× MIC), and 256 μg/mL (16× MIC), respectively. The SI value of PHC was lower than that of ciprofloxacin but comparable to that of tiamulin under the tested conditions.

### 3.3 Safety evaluation of PHC

### 3.3.1 Cytotoxicity assay

The cytotoxicity of PHC was evaluated using a CCK-8 assay in 293T cells, with ciprofloxacin and tiamulin used as reference controls. As shown in **Figure 2D**, PHC exhibited low cytotoxicity across the tested concentration range. At 32 μg/mL, cell viability remained at 90.67%, while at concentrations ≤16 μg/mL, cell viability was maintained at approximately 100% or higher. PHC did not have significant cytotoxic effects on 293T cells under the tested conditions.

### 3.3.2 Haemolysis assay

The haemolytic activity of PHC was evaluated using sheep erythrocytes, and the results are shown in **Figure 2E**. PHC exhibited low haemolytic activity across the tested concentration range (2–512 μg/mL), with no clear concentration-dependent increase in haemolysis. At 32 μg/mL, the haemolysis rate was 6%, while at other concentrations, it remained below 3%. Overall, PHC induced minimal haemolysis of sheep red blood cells under the tested conditions.

### 3.4 Biofilm formation inhibition

PHC inhibited biofilm formation in *Streptococcus suis* SS-27 in a Concentration-dependent manner, as shown by crystal violet staining (**Figure 2F**). Observations (**Figure 2F**) revealed reduced biofilm biomass with increasing PHC concentration.

Quantitative analysis confirmed that PHC had no significant effect at 0.5× MIC (0.0625 μg/mL), whereas significant inhibition was observed at ≥1× MIC (0.125 μg/mL) (P < 0.001), with a further reduction at higher concentrations.

### 3.5 Disruption of cell membrane integrity

**Figure 3:**
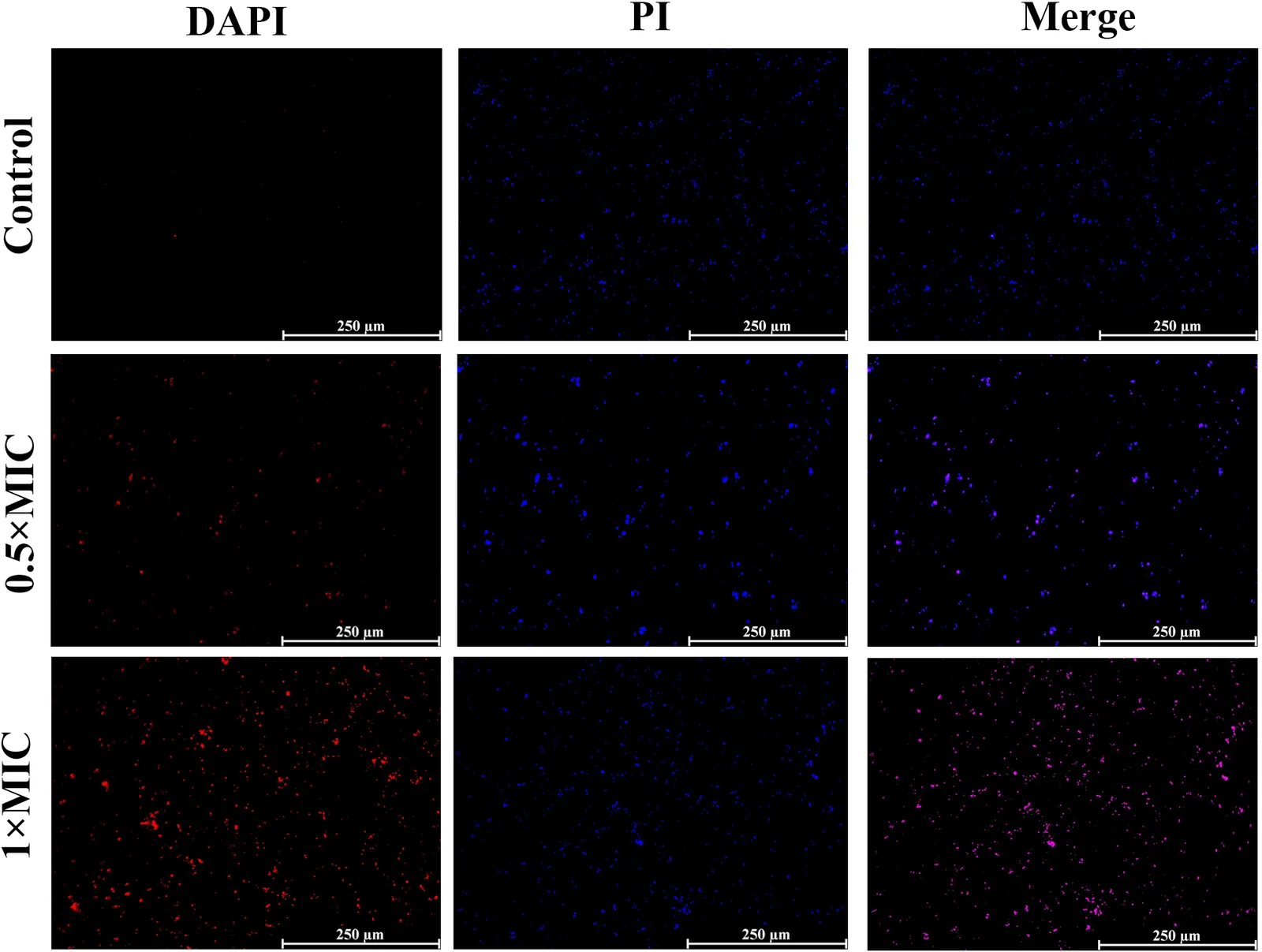
Cell membrane integrity assay.

The cell membrane integrity of *Streptococcus suis* SS-27 was evaluated using DAPI-PI double staining, and representative fluorescence images are shown in **Figure 3**. The control group exhibited predominantly blue fluorescence signals with limited red fluorescence signals, indicating intact cell membranes.

Following treatment with PHC, an increase in red fluorescence intensity was observed. At 0.5× MIC, partial red fluorescence signals appeared, whereas at 1× MIC, red fluorescence signals were widely distributed and accompanied by a marked reduction in blue fluorescence signals, suggesting membrane disruption and cell death.

### 3.6 Cell membrane depolarization

**Figure 4:**
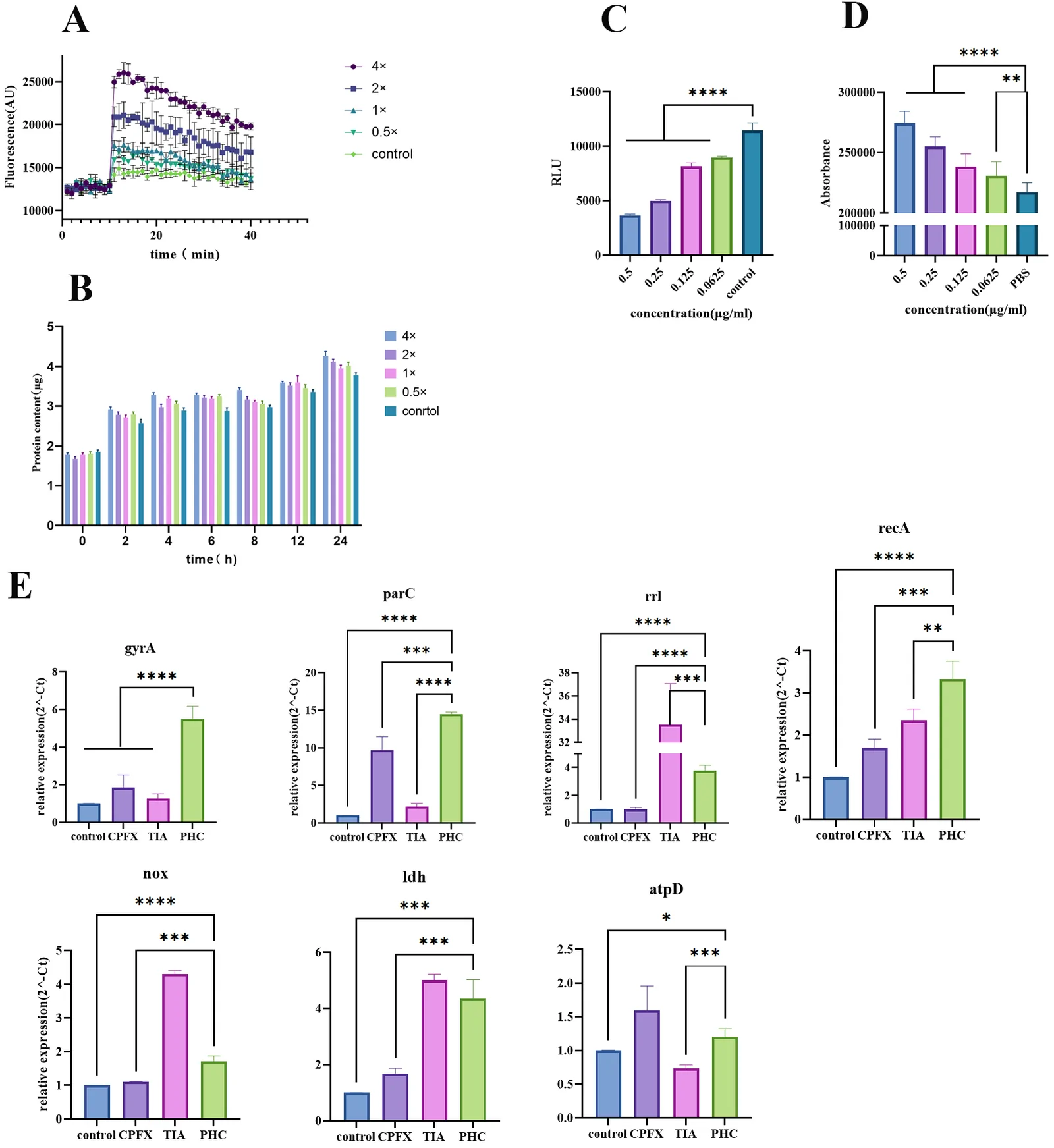
**(A–D)** Effects of different concentrations of PHC on membrane potential depolarization, soluble protein leakage, intracellular ATP levels, and ROS accumulation in *Streptococcus suis* SS-27. **(E)** RT–qPCR. Relative expression levels of target genes in SS-27 after treatment with sub-MIC concentrations of PHC, ciprofloxacin, and tiamulin. All the data are presented as the mean ± SD. p values were determined employing one-way ANOVA and multiple comparisons. ns, p > 0.05; *p < 0.05; **p < 0.01; ***p < 0.001; ****p < 0.0001.

The effects of PHC on the membrane potential of SS-27 cells were assessed using the DiSC3(5) assay, as shown in **Figure 4A**. Prior to PHC addition, the fluorescence intensity remained stable after dye equilibration for 10 min.

Compared with that in the control group, the fluorescence intensity in the PHC-treated group increased significantly. PHC treatment at different concentrations resulted in higher fluorescence signals than those of the untreated control. Overall, higher PHC concentrations were associated with stronger fluorescence responses during the observation period.

### 3.7 Protein leakage assay

Compared with no treatment, PHC treatment increased extracellular protein leakage in *Streptococcus suis* SS-27 (**Figure 4B**). Protein levels in the culture supernatant were consistently higher in the PHC-treated groups than in the control group across all time points.

A concentration-dependent increase in extracellular protein content was observed following PHC exposure.

### 3.8 ATP content assay

As shown in **Figure 4C**, the intracellular ATP levels in *Streptococcus suis* SS-27 decreased with increasing PHC concentration. Significant differences were observed between all treatment groups and the control group (P < 0.0001).

### 3.9 ROS detection assay

As shown in **Figure 4D**, the ROS levels in the PHC-treated groups significantly increased and were positively correlated with the PHC concentration. The 0.5× MIC group exhibited a significant difference compared with the blank group (P < 0.05), while higher concentration groups showed highly significant differences (P < 0.0001).

### 3.10 Regulation of gene expression

The RT–qPCR results are shown in **Figure 4E**. After PHC treatment, the transcript levels of *gyrA*, *parC*, 23S rRNA, *recA*, *nox*, *ldh*, and *atpD* significantly changed.

Compared with those in the control group and the ciprofloxacin group, the transcript levels of *gyrA* and *parC* were significantly upregulated (P < 0.001). DNA gyrase and topoisomerase IV are key enzymes involved in bacterial DNA replication and cell proliferation (Escudero et al., 2007; Hooper and Jacoby, 2016).

23S rRNA is a core component of the ribosomal peptidyl transferase centre responsible for peptide bond formation, and mutations in this region are associated with bacterial resistance (Wilson and Nierhaus, 2003; Pringle et al., 2004; Wilson, 2014). Compared with the control group, the tiamulin group showed a stronger compensatory increase, while the PHC group also exhibited a significant increase (P < 0.001).

*recA* is the key initiator of the bacterial SOS response (Erill et al., 2007). In the PHC-treated group, *recA* expression was significantly increased compared with that in the control group (P < 0.0001), indicating that the bacterial SOS response associated with DNA stress was activated.

*atpD* encodes the β subunit of F_0_F_1_-ATP synthase, which is essential for ATP synthesis and represents a core component of bacterial energy metabolism (Capaldi and Aggeler, 2002). *atpD* expression was significantly upregulated in the PHC group (P < 0.05).

*nox* encodes NADH oxidase (Ahn et al., 2007), which is involved in oxidative stress responses and promotes ROS generation while also affecting glycolysis and NAD^+^ consumption. *nox* expression was significantly upregulated in the PHC group (P < 0.0001).

*ldh* encodes lactate dehydrogenase, which regenerates NAD^+^ to sustain glycolysis and energy production, thereby maintaining the intracellular redox balance (Feldman-Salit et al., 2013). Compared with that in the control group, *ldh* expression significantly increased in both the PHC and tiamulin groups (P < 0.001), with a stronger increase observed in the latter.

### 3.11 Survival rate and therapeutic efficacy

The therapeutic efficacy of PHC was further evaluated using a murine bacteremia model. Following bacterial challenge, infected mice exhibited progressive clinical signs, including roughened fur and body weight loss. The mice in the untreated model group developed generalized tremors and hypothermia, and all the animals died from infection by Day 4 postchallenge.

In the tiamulin-treated group, several mice displayed neurological manifestations, including impaired balance and circling behaviour and reduced mobility. Although these symptoms gradually improved, the affected mice remained unable to maintain a normal posture by Day 7. In contrast, no obvious neurological abnormalities were observed in either the ciprofloxacin- or PHC-treated groups. By Day 7, mice receiving PHC showed marked recovery in activity and coat condition.

**Figure 5:**
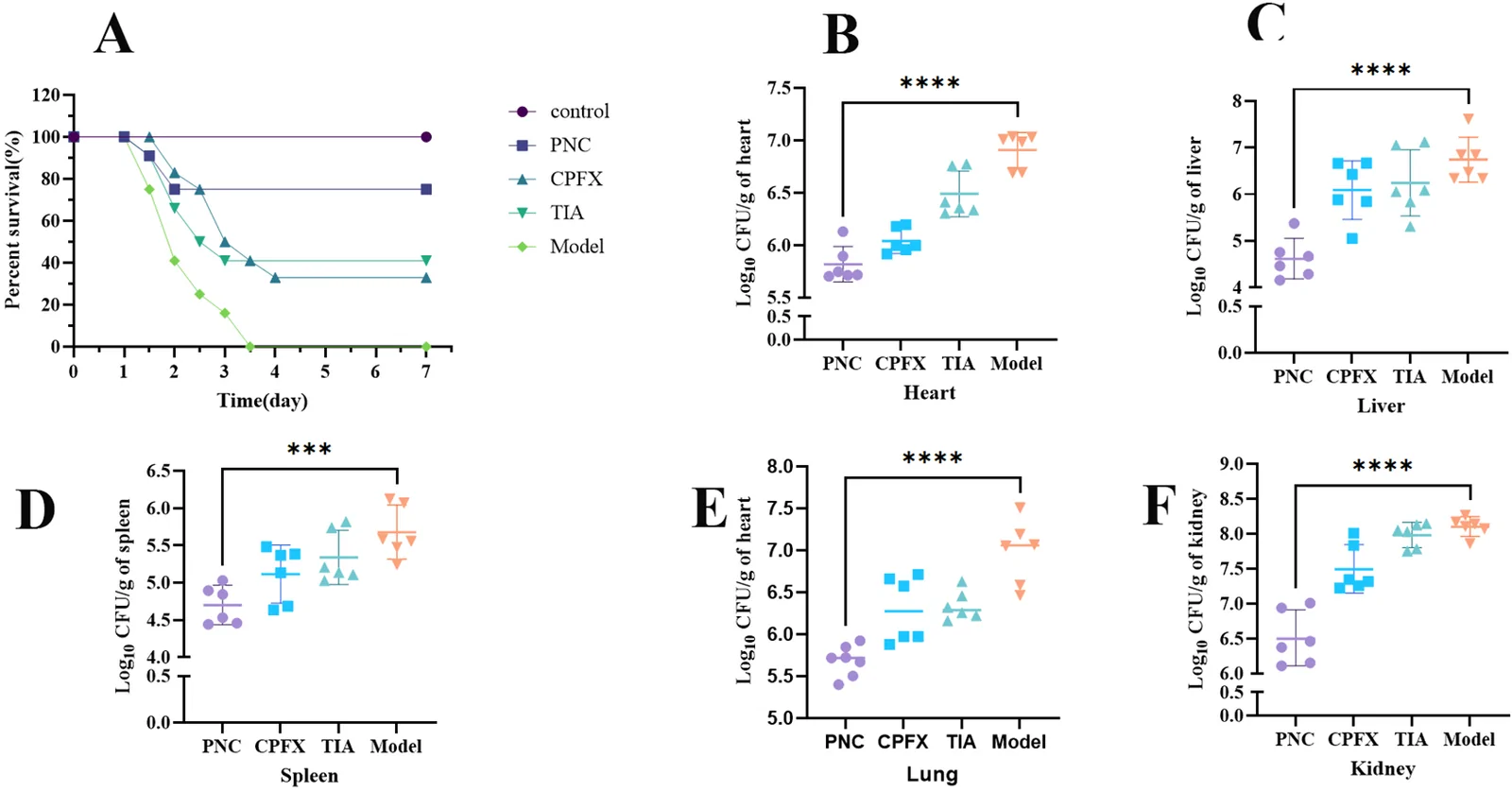
**(A)** Mouse survival rate. **(B–F)** Reduction in bacterial burden in tissues.

Survival outcomes are presented in **Figure 5A**. The PHC-treated group reached a stable survival rate of 75% by Day 2 posttreatment and maintained this level throughout the observation period. In comparison, the tiamulin- and ciprofloxacin-treated groups had four and five surviving mice, respectively, at the end of the experiment. These findings indicate that PHC provided improved protection against *S. suis* infection under the tested conditions.

### 3.12 *In vivo* antibacterial activity – reduction in bacterial burden in tissues

The bacterial burdens in the heart, liver, spleen, lungs, and kidneys of mice with *Streptococcus. suis* bacteremia are presented in **Figures 5B–F**. Compared with the untreated model group, PHC treatment resulted in a reduction of approximately 1–2 log_10_ CFU in all the organs examined. Notably, the decreases in bacterial burden observed in the PHC-treated group were greater than those achieved with ciprofloxacin or tiamulin treatment.

### 3.13 Histopathological evaluation

**Figure 6:**
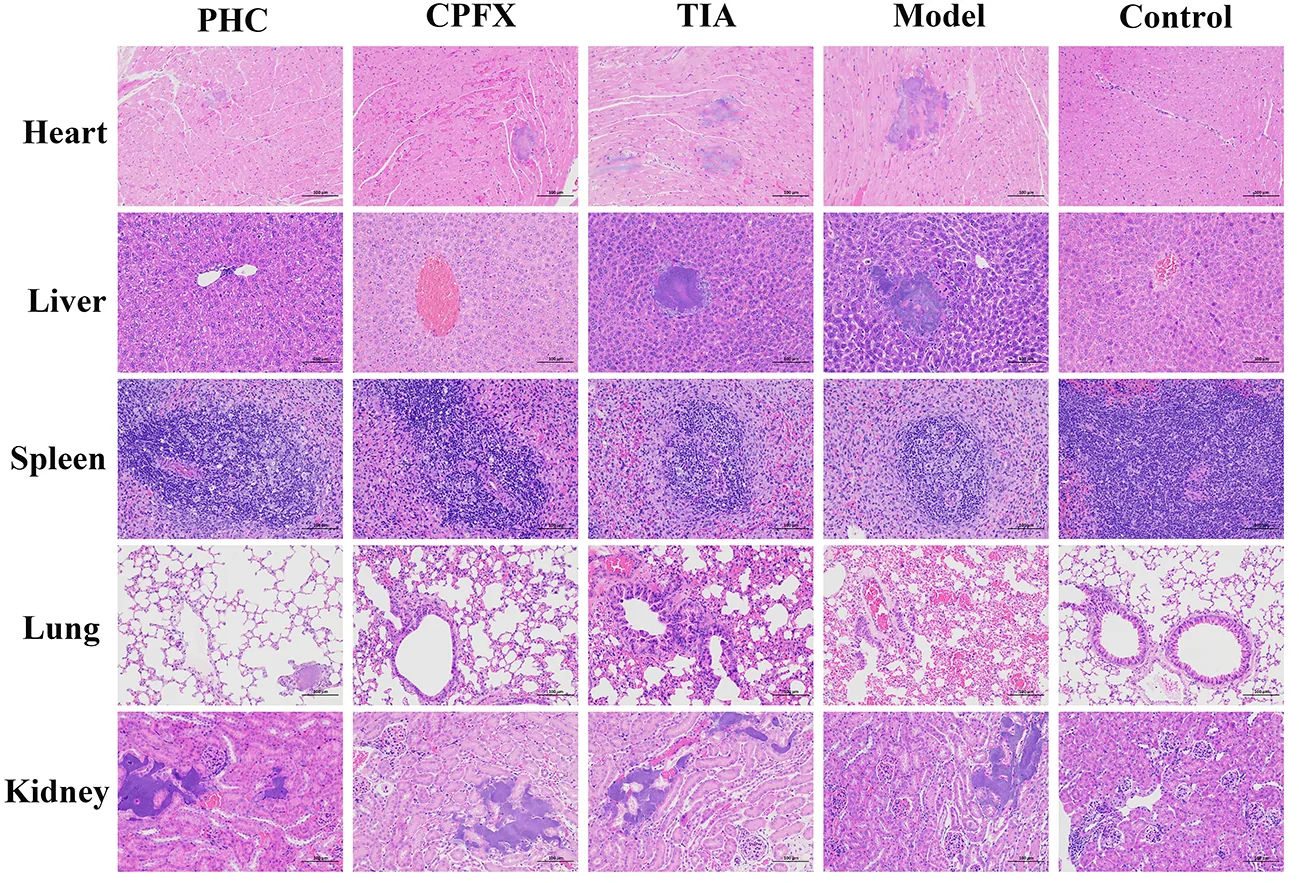
H&E staining images of major organs. The scale bar is 100 μm.

Representative haematoxylin and eosin (H&E)-stained tissue sections are shown in **Figure 6**. No obvious histopathological abnormalities were observed in the heart, liver, spleen, lungs, or kidneys of the blank control group.

In contrast, mice in the untreated model group exhibited varying degrees of tissue injury. Cardiac tissue showed focal inflammatory cell infiltration accompanied by focal myocardial necrosis and nuclear pyknosis. Liver sections exhibited cytoplasmic vacuolation, focal necrotic lesions within the hepatic sinusoids, hepatocellular degeneration with nuclear pyknosis, and vascular congestion. Splenic tissue displayed focal lymphocyte necrosis, nuclear pyknosis and fragmentation, and mononuclear cell infiltration. The lung tissue showed granulocyte infiltration within the alveolar walls, diffuse thickening of the alveolar septa, focal alveolar haemorrhage, and mild bronchial epithelial desquamation. Renal sections revealed degeneration, necrosis, and desquamation of tubular epithelial cells, accompanied by nuclear pyknosis and eosinophilic cytoplasmic changes.

Compared with those in the untreated model group, histopathological lesions in the PHC-, ciprofloxacin-, and tiamulin-treated groups were alleviated to varying degrees. Among these treatment groups, the PHC group exhibited the most pronounced improvement, with reduced inflammatory infiltration, tissue degeneration, and necrotic lesions across multiple organs.

### 3.14 Predicted binding sites

**Figure 7:**
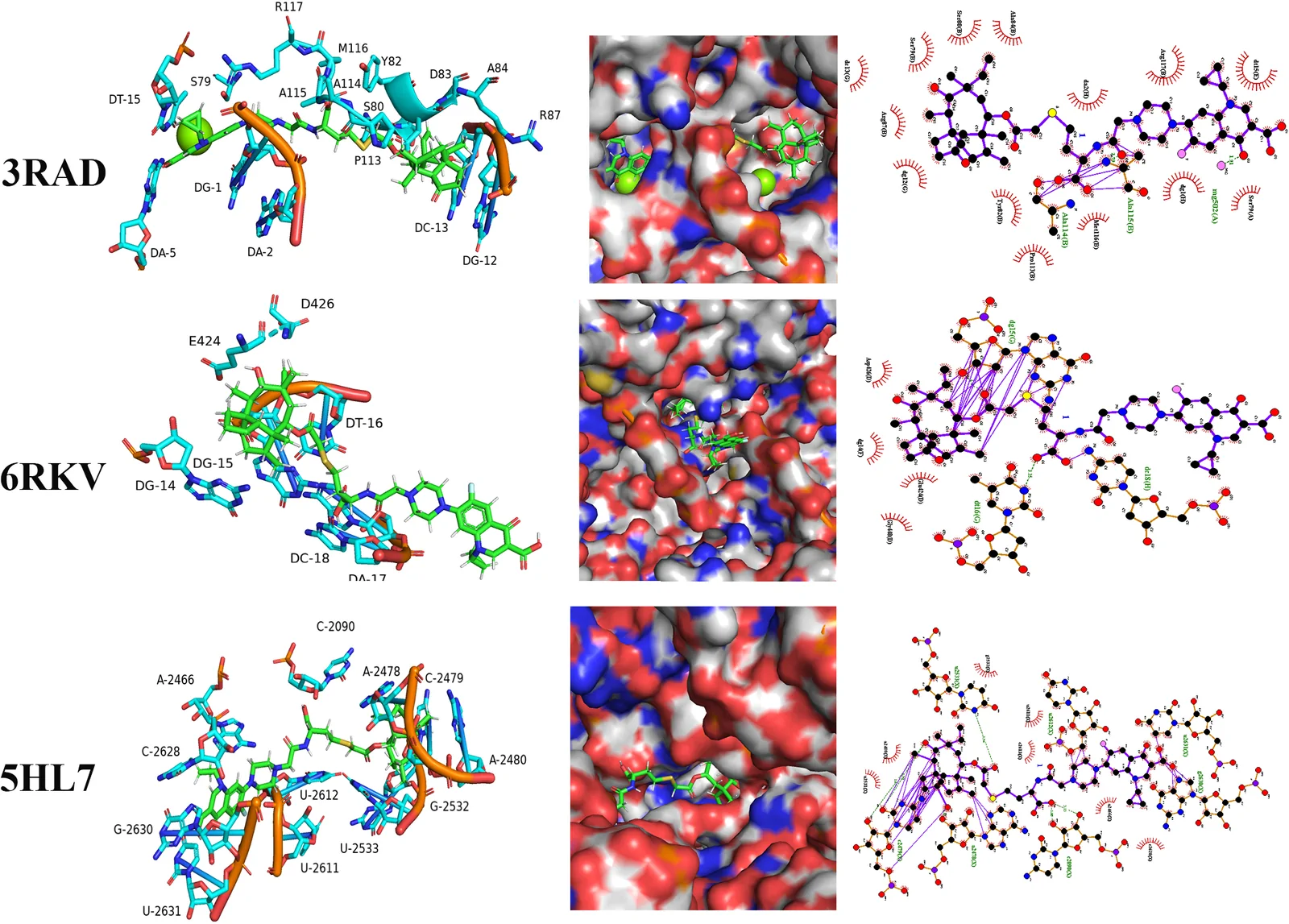
Molecular docking prediction of interactions between PHC and 3RAD, 6RKV, and 5HL7.

As **Figure 7** shows, molecular docking analysis demonstrated that PHC could stably bind to the active pocket of 3RAD, with a binding affinity of −7.5 kcal/mol. PHC formed hydrogen bonds between its O3 and O4 atoms and Ala115(B) and Mg502(A), with bond lengths of 2.79 Å and 1.91 Å, respectively. The short hydrogen bond distances suggest stable ligand–target interactions. In addition, PHC established multiple hydrophobic interactions with residues, including Arg117, Met116, Tyr82, and Pro113, facilitating its accommodation within the binding cavity. Collectively, these interactions indicate favourable binding compatibility between PHC and 3RAD.

PHC also exhibited stable binding to the 6RKV target, with a binding energy of −7.6 kcal/mol. Docking conformations indicated that PHC was stabilized within the binding pocket through hydrogen bonding, hydrophobic interactions, and extensive π-related interactions. Compared with 3RAD, the interaction profile in 6RKV was more dependent on aromatic stacking and hydrophobic contacts. Nucleic acid residues, including dg15, dc18, and dt16 contributed to the formation of hydrogen bonds with PHC, thereby increasing complex stability. In addition, abundant π interactions involving the aromatic scaffold of PHC further supported its stacking stability within the binding environment. Surface analysis confirmed that PHC was well embedded within the binding cavity, indicating high spatial complementarity. These findings support a potential binding interaction between PHC and 6RKV.

PHC showed the strongest binding affinity towards 5HL7, with a binding energy of −10.0 kcal/mol, suggesting a stable ligand–target interaction. Structural analysis revealed that PHC was embedded within the binding pocket and formed an extensive interaction network with surrounding nucleotides. Multiple hydrogen bonds were observed between PHC and U2612, U2533, A2478, and C2090, with bond lengths of approximately 2.0–2.4 Å, contributing to enhanced binding stability. Furthermore, the aromatic moiety of PHC engaged in extensive π-related interactions with nucleobases within the pocket, potentially reinforcing binding stability via π–π stacking. Additional hydrophobic interactions further stabilized the ligand within the cavity. Surface representation confirmed the strong spatial complementarity between PHC and the binding pocket. Overall, PHC demonstrated strong binding affinity towards 5HL7 and may interfere with the function of the 50S ribosomal subunit.

## 4 Discussion

Redesigning existing antibiotics represents a promising strategy to circumvent resistance mechanisms developed by pathogenic bacteria. Hybrid antibiotic approaches aim to overcome established resistance by simultaneously or sequentially targeting two distinct sites in pathogens (Pokrovskaya and Baasov, 2010). In this context, the design of PHC was guided by several objectives, including improving antibacterial efficacy compared with that of the parent compounds (truncated side-chain derivatives and ciprofloxacin), enhancing *in vivo* antibacterial potential, and expanding the antibacterial spectrum derived from the parent molecules. In addition, the hybrid design was intended to reduce the likelihood of resistance emergence associated with single-target mutations through a dual-target strategy.

In *in vitro* antibacterial assays, compared with ciprofloxacin and tildipirosin, PHC exhibited markedly greater activity against *Streptococcus suis*. On the basis of broth microdilution testing, the MIC of PHC was 32-fold lower than that of tildipirosin and 64-fold lower than that of ciprofloxacin. PHC also effectively inhibited the growth of drug-resistant *S. suis*. Notably, the antibacterial effect showed limited enhancement with increasing concentrations, suggesting a time-dependent killing pattern.

Antibiotic misuse is a major driver of bacterial resistance; therefore, dosing strategies that do not rely on high drug concentrations may help reduce the risk of resistance development.

PHC is a novel hybrid molecule generated by conjugating a truncated pleuromutilin derivative with ciprofloxacin through an L-homocysteine linker, thereby integrating the structural features of both parent compounds. In the present study, the antibacterial mechanism of PHC was preliminarily investigated through molecular docking, RT–qPCR, and a series of functional assays.

PHC treatment disrupted the integrity of the *Streptococcus suis* cell membrane and induced membrane depolarization, thereby impairing membrane barrier function and membrane potential homeostasis. The resulting increase in membrane permeability facilitated the leakage of intracellular constituents, which may contribute to bacterial cell death. Membrane depolarization has been reported to promote intracellular ROS accumulation (Gray et al., 2024), and consistent with this observation, PHC treatment was associated with elevated ROS levels and a marked consumption of intracellular ATP.

Excessive ROS accumulation may further impair central metabolic processes, including glycolytic activity and DNA synthesis, ultimately leading to irreversible cellular damage (Vaishampayan and Grohmann, 2021). In response to these stresses, the expression of genes involved in energy metabolism and redox regulation is altered in *S. suis*, suggesting that adaptive metabolic responses are aimed at maintaining cellular homeostasis under PHC-induced stress.

Quinolones primarily exert their antibacterial activity by targeting DNA gyrase in gram-negative bacteria and topoisomerase IV in gram-positive bacteria, both of which are essential enzymes responsible for maintaining DNA topology during replication and cell division (Escudero et al., 2007; Hooper and Jacoby, 2016). In the present study, RT–qPCR analysis revealed significant upregulation of *gyrA* and *parC* following PHC treatment. Because bacteria frequently increase the expression of genes encoding topoisomerases in response to impaired DNA topology and replication stress, these transcriptional changes may reflect a compensatory response to PHC-induced interference with DNA replication processes.

Consistent with the transcriptional data, molecular docking analysis demonstrated favourable binding affinities of PHC towards both the 3RAD and 6RKV complexes, suggesting its potential to interact with DNA topoisomerase IV and DNA gyrase. Notably, quinolone resistance in *Streptococcus suis* is largely associated with point mutations within the quinolone resistance-determining regions (QRDRs) of the GyrA subunit of DNA gyrase and the ParC subunit of topoisomerase IV (Escudero et al., 2007; Hooper and Jacoby, 2016). The observed interactions between PHC and these targets indicate that PHC retains the capacity to engage key enzymes involved in DNA replication and support its potential as a candidate for further investigation against quinolone-resistant *Streptococcus suis*.

23S rRNA constitutes the catalytic core of the 50S ribosomal subunit and serves as the primary binding region for pleuromutilin antibiotics (Pringle et al., 2004). In the PHC-treated group, 23S rRNA expression was significantly upregulated, although the magnitude of this compensatory response was lower than that observed following tiamulin treatment. Molecular docking analysis revealed that PHC exhibited slightly stronger binding affinity towards the 50S ribosomal complex (5HL7) than towards either 3RAD or 6RKV, suggesting that the bacterial ribosome may represent an equally important target of PHC. The relatively modest increase in 23S rRNA expression, compared with the more pronounced upregulation of *gyrA* and *parC*, may indicate that the bacterial compensatory response was preferentially directed towards maintaining DNA topology and replication under PHC-induced stress.

As a central regulator of the bacterial SOS response, *recA* was significantly upregulated following PHC treatment, indicating the induction of DNA replication stress or DNA damage responses. In addition, PHC significantly increased the expression of *nox* and *ldh*, two genes involved in cellular redox regulation and energy metabolism. *nox* encodes NADH oxidase, which contributes to oxidative stress responses through modulation of the intracellular redox balance, whereas *ldh* participates in NAD^+^ regeneration to sustain glycolytic activity and energy production. Consistent with these transcriptional changes, intracellular ROS levels were markedly elevated after PHC exposure. Collectively, these findings suggest that PHC induces substantial oxidative stress and triggers adaptive metabolic responses in *Streptococcus suis*.

**Figure 8:**
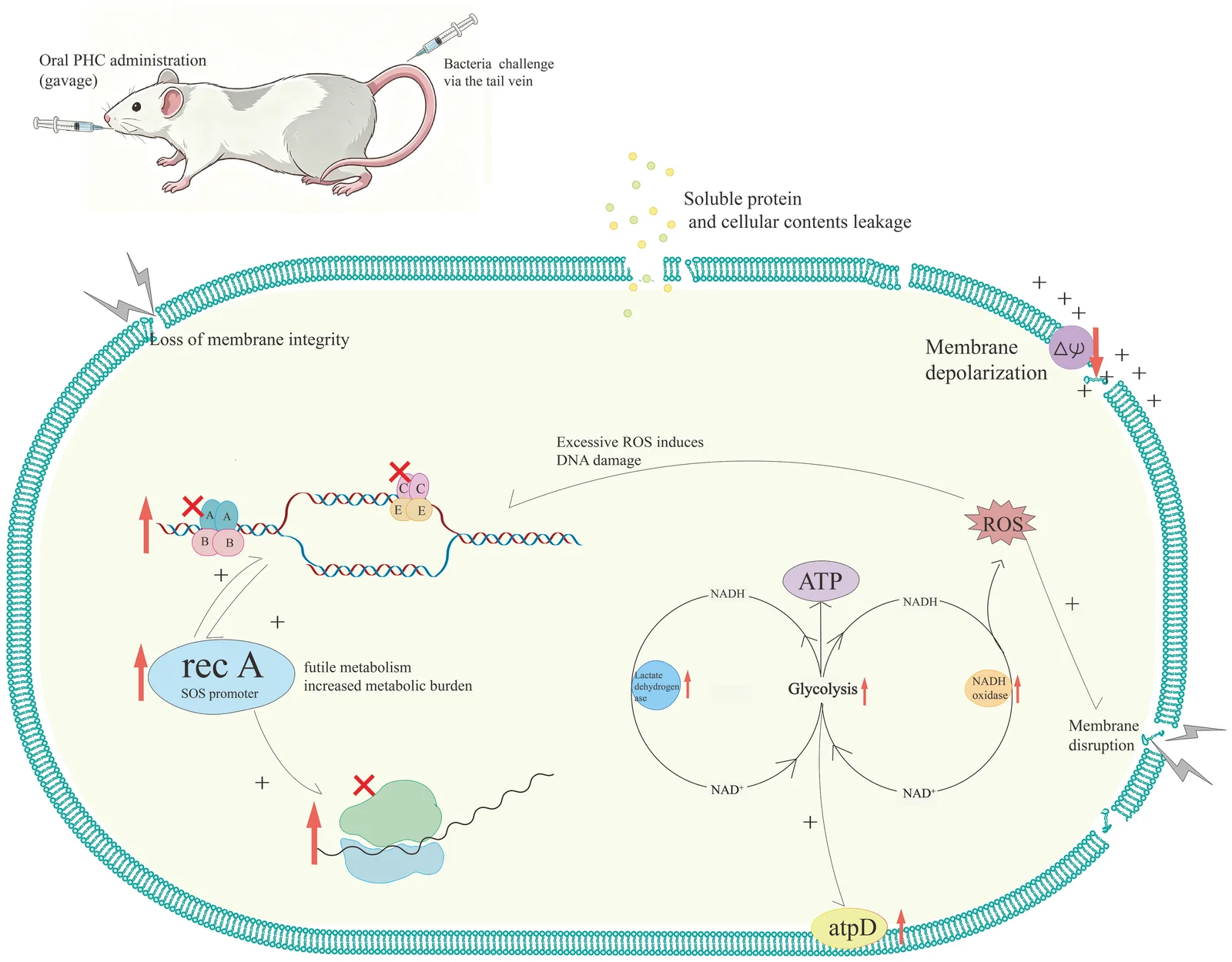
PHC interferes with DNA replication- and protein synthesis-associated pathways, accompanied by transcriptional upregulation of genes related to DNA gyrase, topoisomerase IV, and 23S rRNA. PHC-induced DNA damage activates recA expression, driving futile DNA repair and protein synthesis. PHC upregulates atpD increasing ATP production to support futile compensatory responses. *nox* and *ldh* are upregulated to facilitate NADH turnover and maintain redox homeostasis under PHC-induced oxidative stress, while Nox-dependent oxidation promotes ROS accumulation. PHC induces membrane depolarization and compromises membrane integrity, resulting in the leakage of soluble proteins and intracellular contents and ultimately accelerating bacterial death.

In addition to *ldh*, PHC treatment significantly upregulated *atpD*, which encodes the β subunit of the F_0_F_1_-ATP synthase and plays a central role in bacterial ATP production. Consistent with these transcriptional changes, intracellular ATP levels were markedly reduced following PHC exposure. One possible explanation is that PHC-induced stress responses increase the energy demands required to maintain cellular homeostasis and counteract oxidative damage. Although the upregulation of *atpD* may reflect an attempt to increase ATP synthesis, the substantial decrease in intracellular ATP suggests that energy consumption exceeded ATP production under PHC treatment. Collectively, these findings indicate that PHC disrupts bacterial energy homeostasis and imposes a considerable metabolic burden on *Streptococcus suis*. In addition to conventional antimicrobial agents, recent biomaterial-based strategies have been explored for managing infections and oxidative stress in livestock. For instance, ROS-scavenging hydrogels have been developed to prevent postoperative adhesions (Li et al., 2024; Pan et al., 2025), and dual-layer antibacterial hydrogels have shown synergistic effects against bacterial wound infections (Cai et al., 2023; Luo et al., 2025). In contrast to these ROS-eliminating or material-based approaches, the authors hybrid molecule PHC exerts direct bactericidal activity through intrinsic ROS induction and dual-target disruption, offering a complementary chemical strategy for combating S. suis infections in swine production. **Figure 8** illustrates mechanisms mentioned above.

In a murine infection model, PHC demonstrated favourable therapeutic efficacy. Compared with the thiamulin- and ciprofloxacin-treated groups, the PHC group achieved greater reductions in bacterial burdens across the heart, liver, spleen, lungs, and kidneys. Consistent with these findings, PHC treatment was associated with alleviated clinical manifestations, improved survival outcomes, and reduced severity of histopathological lesions in multiple organs. Collectively, these results indicate that PHC effectively mitigated the progression of *Streptococcus suis* infection *in vivo*.

PHC exhibited a favourable safety profile in both the CCK-8 cell viability assay and the haemolysis assay. In the CCK-8 test, PHC showed low cytotoxicity towards mammalian cells within the tested concentration range, with no significant reduction in cell viability observed at the evaluated doses. Consistently, in the haemolysis assay, PHC induced negligible haemolytic activity against red blood cells across the tested concentrations, indicating minimal disruption of erythrocyte membrane integrity. Collectively, these results suggest that PHC possesses good *in vitro* biocompatibility and a favourable safety profile for further evaluation.

Several limitations of this study should be acknowledged. First, the time–kill assays demonstrated that PHC at 1×, 2×, and 4× the MIC produced comparable antibacterial effects, whereas a marked reduction in activity was observed at 0.5× the MIC. The basis for this apparent plateau in bactericidal activity remains unclear and warrants further investigation. Second, although PHC significantly reduced biofilm biomass, the present study could not distinguish whether this effect resulted primarily from the inhibition of biofilm formation or from a reduction in bacterial growth and viability. Additional experiments specifically targeting biofilm development are therefore needed.

Based on the transcriptional responses and the molecular docking analyses, PHC may exert antibacterial activity through potential dual actions involving topoisomerase-related proteins and ribosome-associated components. Direct target validation studies, including cellular thermal shift assays (CETSAs), drug affinity-responsive target stability (DARTS) assays, and enzyme activity inhibition assays, are required to further confirm these potential interactions.

## Data Availability

The original contributions presented in the study are included in the article. Further inquiries can be directed to the corresponding author.
